# The Relationship of Comorbidities with Intensive Care Unit Admission and Mortality in Patients with COVID-19

**DOI:** 10.5152/TJAR.2021.21058

**Published:** 2022-06-01

**Authors:** Büşra Yıldız, Seyfettin Erden, Ahmet Öz, Turgut Karabağ

**Affiliations:** 1 Department of Coronary Care Unit, İstanbul Education and Research Hospital, İstanbul, Turkey; 2 Department of Cardiology, İstanbul Education and Research Hospital, İstanbul, Turkey

**Keywords:** Charlson Comorbidity Index, COVID-19, exitus, intensive care, prognosis, respiratory support

## Abstract

**Objective:**

Comorbid conditions are known to be associated with poor prognosis in coronavirus disease 2019. This study aimed to investigate the effects of comorbidity burdens of inpatients, identified by the Charlson Comorbidity Index, on their mortalities.

**Methods:**

A total of 150 patients who presented to the emergency department of our hospital with various complaints and symptoms were diagnosed with coronavirus disease 2019 as a result of the testing and received inpatient treatment (87 males, mean age 61.6 ± 13.8 years) were included in the study. Charlson Comorbidity Index scores were calculated. Patients were classified into 2 groups based on the state of exitus: group 1, those who did not survive; 33 patients, 19 males; 68.3 ± 11.8 years and group 2, those who survived; 117 patients, 68 males; 59.7 ± 13.8 years.

**Results:**

In all patients, the exitus rate was 22%, the rate of intensive care follow-up was 46%, and the intubation rate was 37.3%. The Charlson Comorbidity Index scores were significantly higher in group 1 compared to group 2. Multivariate logistic regression analyses demonstrated that the Charlson Comorbidity Index score was an independent predictor of in-hospital mortality (odds ratio: 1.990, 95% CI: 1.314-3.015, *P*  = .001). The cut-off value for the Charlson Comorbidity Index to predict in-hospital mortality was 5.5, with 81.8% sensitivity and 73.5% specificity.

**Conclusions:**

The Charlson Comorbidity Index score, which can be obtained at the time of admission, could be associated with the prognosis of coronavirus disease 2019 patients. Those with a Charlson Comorbidity Index score greater than 5.5 could be more associated with negative outcomes and mortality.

## Main Points

- Comorbid conditions are associated with poor prognosis.
- Charlson Comorbidity Index obtained at the time of admission could be associated with the prognosis of coronavirus disease 2019 patients.
- Charlson Comorbidity Index score of greater than 5.5 could be more associated with negative endpoints and mortality.

## Introduction

The coronavirus disease 2019 (COVID-19) belongs to a large family of viruses known to cause diseases from simple colds to Middle East respiratory syndrome and acute respiratory distress syndrome.^1-3^ Most infections are not severe; fever and cough are the most common symptoms.^4^ Individuals with mild to moderate COVID-19 symptoms can be treated with general isolation, and they do not need intensive care if their condition does not get worse.^5^ However, critical conditions such as respiratory failure, multiple organ failure, and shock have been reported in 5% of patients.^6^ Determining the risk profiles of these patients in the early stages of the disease would be important in predicting the patients whose general condition would get worse during follow-up. Therefore, early prognosis predictions can help reduce mortality and shortage of medical resources.^5^

The Charlson Comorbidity Index (CCI) is a scale for the comorbidity burdens of patients and is a prognostic indicator used in measuring the prognostic effects of 22 different medical conditions.^7^ It has been widely used to evaluate comorbidities in different populations.^8-12^

The aim of the study is to investigate the effects of comorbidity burdens detected by the CCI on the need for follow-up and/or respiratory support in the intensive care unit and the mortality rates of patients diagnosed with COVID-19.

## Methods

### Patient Population

A total of 150 patients who presented to the emergency department of our hospital with various complaints and symptoms were diagnosed with COVID-19 as a result of the testing and received inpatient treatment (87 males and 63 females; mean age 61.6 ± 13.8 years) were included in the study. Detailed past medical history and physical assessments were performed and the information was recorded. The patients’ histories were taken in detail; acute and chronic disorders experienced by the patients in the past and present were recorded. Their temperatures were measured, and their pulses, respiratory counts, systolic-diastolic blood pressures, and oxygen saturations obtained from fingertips via pulse oximetry were recorded. Venous blood samples were taken from all patients; hemogram, D-dimer, liver and kidney functions, C-reactive protein (CRP), troponin, and lactate dehydrogenase (LDH) values were measured at the time of admission. 

Chest computed tomography was taken for all patients through posteroanterior chest radiography. Patients were diagnosed with COVID-19 according to the diagnostic plan of the Republic of Turkey, Ministry of Health, National Science Committee.^13^ The diagnostic criteria were having at least one of the signs and symptoms of fever or acute respiratory disease (cough and respiratory distress), an inability to explain the clinical picture by another cause/disease, a history of being abroad or having a relative who was abroad within 14 days before the symptoms started, or identification of severe acute respiratory syndrome coronavirus 2 obtained from patients with confirmed COVID-19 cases through molecular methods. In addition to clinical manifestations, characteristic findings of COVID-19 pneumonia obtained via imaging methods reduced the number of lymphocytes despite normal or reduced leukocyte counts at the laboratory, and a high amount of serum-reactive protein was also considered supporting findings. 

The criteria for hospitalization were based on the observation of fever, cough, dyspnea, tachypnoea, hypoxemia, hypotension, widespread radiological findings in the lungs, and changes in consciousness in a patient with acute respiratory disease developed within the last 14 days. 

Patients were treated for COVID-19 based on the treatment plan issued by the Republic of Turkey, Ministry of Health, National Science Committee.^14^ Hydroxychloroquine ± azithromycin, favipiravir, and/or lopinavir/ritonavir treatments were administered to the patients according to their clinical conditions and findings. 

Among the inpatients, those who developed respiratory failure, who needed respiratory support or mechanical ventilation, and who experienced shock and multiple organ failure were taken to the intensive care unit. 

The total number of hospitalization days was recorded. An informed consent form was obtained from all patients. The study was approved by the local ethics committee with the ethical committee decision numbered 1245 in May 2020. 

### Charlson Comorbidity Index

Charlson Comorbidity Index is a questionnaire used for the mortality assessment model in which the varying impact of several chronic conditions in 1-year mortality is considered. Necessary information to calculate the index was obtained through history-taking at the time of admission. It was calculated by the sum of the comorbidity scores of each condition.^7^ The score was calculated for each participant. It was also classified as mild (scores 1-2), moderate (scores 3-4), and severe (score ≥5).

### Statistical Analysis

Statistical Package for the Social Sciences 22.0 software package (IBM Corp.; Armonk, NY, USA) was used for analyzing the study data. The numerical variables were expressed as the mean ± standard deviation, and non-normal distributed variables were expressed with the median. Categoric variables were expressed as frequency (n) and percentage (%). Kolmogorov–Smirnov test was used for testing the normality of the distribution of numerical variables. Independent samples *t*-test and Mann–Whitney *U* test were used for the comparison of continuous variables between 2 independent groups whichever is suitable for the data. Pearson correlation analysis was used to evaluate the relevance between the CCI score and several parameters. To assess the independent contribution of each variable, a multiple logistic regression analysis that included all clinical variables with a *P* < .05 in the univariate analysis was performed. A receiver operating characteristic curve analysis was used to find out the CCI score value that predicted mortality with the best specificity and sensitivity. A *P* value of less than .05 was considered statistically significant. 

## Results

Patients were classified into 2 groups based on the state of exitus: group 1, those who did not survive; 33 patients, 19 males, 14 females; 68.3 ± 11.8 years and group 2, those who survived; 117 patients, 68 males, 49 females; 59.7 ± 13.8 years. 

For all patients included in the study, the exitus rate was 22%, the intensive care follow-up rate was 46%, and the intubation rate was 37.3%. The number of patients with mild CCI scores was 28, the moderate score was 43, and the high score was 79.

In group 1, age and the incidence of diabetes, coronary artery disease, and kidney failure were higher compared to group 2 (Table 1). The CCI score was significantly higher in patients with exitus compared to the patients without (7.6 ± 2.6 vs 4.1 ± 2.2, *P* < .001). All biochemical values, except for D-dimer and troponin, were significantly higher in group 1 compared to group 2 (Table 1). Those who died had a higher white blood cell count, and their platelet and lymphocyte cells were significantly lower (Table 1). The neutrophil-to-lymphocyte ratio (NLR) was significantly higher in group 1 compared to group 2 (29.6 ± 45.5 vs 9.1 ± 38.4, *P*  = .011).

When we classified according to the CCI score degree, age, length of stay in the hospital, number of patients with coronary artery disease, hypertension, diabetes mellitus, chronic renal disease, respiratory rate, oxygen saturation, white blood cell, neutrophil, glucose, urea, creatinine, LDH, CRP, favipiravir use were significantly higher in patients with high CCI score compared to patients with mild and moderates (Table 2). 

Correlation analysis resulted in a negative correlation with CCI score and oxygen saturation and a positive correlation with white blood cell, LDH, CRP, and respiratory rate (Table 3). 

Univariate and multivariate logistic regression models associated with hospital mortality are presented in Table 4. Urea, CCI, white blood cell, alanine transaminase, aspartate transaminase, LDH, CRP, glucose, creatinine, oxygen saturation, in-hospital mortality, days of hospitalization, platelet and lymphocyte count, and favipiravir use were found to be the determinants. Multivariate logistic regression analyses were performed to identify independent predictors of hospital mortality using variables that demonstrated marginal association with in-hospital mortality in univariate analyses. 

**Figure 1.**
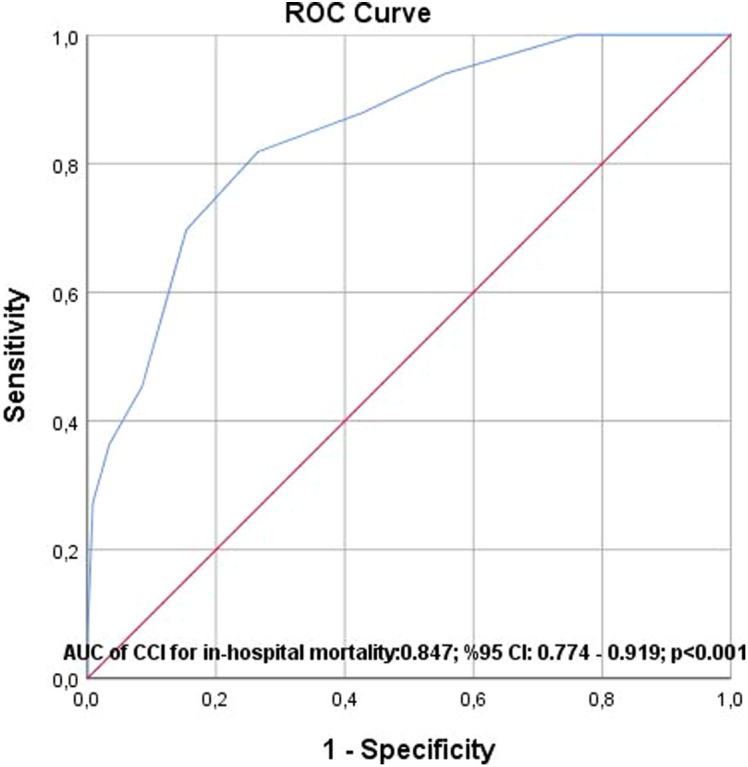
ROC curve of CCI used to predict exitus in patients with COVID-19. ROC, receiver operating characteristic; CCI, Charlson Comorbidity Index; COVID-19, coronavirus disease 2019.

In multivariate logistic regression analysis, oxygen saturation, heart rate, blood urea nitrogen, basal serum creatinine level, aspartate transaminase, and CCI were found to be independent predictors of hospital mortality (Table 4). Receiver operating characteristic analysis concluded that the area under the curve values for CCI in terms of in-hospital mortality was 0.847 (95% CI: 0.774-0.919, *P* < .001; Figure 1). The threshold for predicting in-hospital mortality for the CCI was 5.5, with 81.8% sensitivity and 73.5% specificity.

## Discussion

The main conclusion of our study is that the CCI is an index related to mortality in patients with COVID-19 and is a parameter that should be considered regarding the need for intensive care and respiratory support. The scores of individuals who resulted in mortality or who required respiratory support were significantly higher. In these patients, the CCI score was associated with laboratory and physical examination parameters demonstrating the severity of the disease. The CCI score to be determined during the admission of patients with COVID-19 could be important in the follow-up and treatment processes.

Since its outbreak at the end of 2019,^15-17^ COVID-19 has caused infections and pandemic in many countries of the world. It has been observed to be more contagious and severe than the factors that cause many known respiratory infections.^17^ The disease progresses with severe respiratory symptoms and high mortality. In their compilation study on the data obtained from 20 different regions during the pandemics in Italy, Immovili et al^18^ explained the mortality rate as 7.5% (3.1%-16.7%) and the rate of need for intensive care as 21.4% (9.4%-45.9%). Among the patients with COVID-19 infections who were hospitalized in our hospital, the mortality rate and the rate of need for intensive care were higher compared to these findings. 

Wang et al^4^ examined the demographic and clinical profiles of 138 patients hospitalized in Wuhan with pneumonia caused by COVID-19. The number of patients requiring follow-up in the intensive care unit was 36, and the average age of these patients was higher than other patients (66 years vs 51 years). Again, more comorbidity was present in patients followed up in the intensive care unit (72.2% vs 37.3%). The mortality was found to be 4.3%.

In their analysis of 72 314 COVID-19 cases, 62% of who were confirmed, Wu et al.^6^ found that the vast majority of the cases were aged between 30 and 79, and 3% were over 80 years of age. They found that 14% of the cases experienced severe disease and 5% were in a critical stage. It was found that mortality developed in 2.3% of the confirmed cases. Among these cases of mortality, 24.8% were identified to be above the age of 70 years. They showed that the presence and frequencies of comorbid conditions in cases with mortality are remarkable.

In another study conducted in China, 34 patients requiring intensive care treatment were analyzed; these patients were divided into 2 groups according to non-invasive and invasive ventilation needs. It was found that patients who needed invasive mechanical ventilation had lower lymphocyte counts and higher blood urea nitrogen, LDH, platelet, D-dimer, and hemoglobin levels, as well as more complications.^19^ In our study, CRP, LDH, urea, creatinine, aspartate aminotransferase (AST), alanine aminotransferase (ALT), and troponin were significantly higher in individuals with negative outcomes and in individuals who resulted in mortality.

In a multicenter study, Bajaj et al^20^ compared the outcome among the age/gender-matched patients with cirrhosis + COVID-19 versus patients with COVID-19 alone and cirrhosis alone. They concluded that patients with cirrhosis + COVID-19 had similar mortality compared with patients with cirrhosis alone. They also found cirrhosis + COVID-19 had higher mortality than patients with COVID-19 alone. Also, they found CCI was the only independent mortality predictor among the study group.

Imam et al^21^ investigated mortality predictors of COVID-19 in a large cohort of hospitalized patients in the United States in a retrospective study. Among 1305 patients who were hospitalized during the evaluation period, the median CCI was 2,^1-4^ 72.6% and multivariates regression analysis revealed that CCI > 3 (OR: 2.71, 95% CI: 1.85-3.97) were independently associated with mortality^21^ In our study, patients with exitus had a mean CCI score of 7.6 ± 2.6 and it was one of the predictors of in-hospital mortality in our study. 

In our study, the age was higher in patients who needed intensive care and respiratory support and who died. The drugs given to both groups were similar; however, it was observed that the group with less mortality received more favipiravir. It was observed that kidney functions were worse, CRP levels were higher, and lymphocyte levels were lower in patients who resulted in mortality and resulted negatively. The CCI score was significantly higher in patients with mortality and adverse outcomes. The CCI score and respiratory rate were associated with oxygen saturation. In regression analysis, the most relevant parameter to mortality was the CCI score. A CCI score above 5.5 could be valuable in predicting mortality. 

In a study that prospectively included 61 patients with COVID-19 infection as a derivative cohort and 54 patients as a validation cohort, Liu et al^5^ demonstrated that the NLR was an important parameter in predicting cryptic disease. They concluded that the disease has become more critical and there is a greater need for intensive care, especially in patients over the age of 50 with an NLR > 3.13. In our study, lymphocyte values were significantly lower in those who reached the endpoint and died. The NLR was significantly higher only in those who died compared to those who were not. However, we were not able to identify any correlation between death, lymphocyte count, and NLR in regression analysis.

At the time of the study, the ministry of health in the country where the study was conducted, hydroxychloroquine was used and recommended in the treatment of COVID-19. Nowadays, new developments are experienced every day in the diagnosis and treatment of COVID-19. Currently, hydroxychloroquine has been discontinued in our country in the treatment of COVID-19. Hydroxychloroquine is a drug that may affect cardiac mortality. Therefore, hydroxychloroquine may have an effect on mortality in the current study. Since the same drugs were given to all patient groups, we think that the effect of the study was not significant.

## Conclusion

In our study, we used the CCI score, which indicated the comorbidity burden of the patient cumulatively, instead of the risk factors or concomitant diseases to be associated with mortality and intensive care needs in each patient separately. The score, which could easily be obtained at the time of admission, could be associated with the prognosis of COVID-19 patients. Those with a CCI score of greater than 5.5 could be more associated with negative endpoints and mortality. Certainly, respiratory status and hemodynamics of patients at the time of admission or follow-up would be important regarding the need for intensive care. In addition to these, we believe that a multidisciplinary approach besides medical treatment and respiratory support applied to individuals with a higher comorbidity burden could be more rational in these patients.

## Figures and Tables

**Table 1.** Demographic Features, Vital Signs, Laboratory Findings, and the Medication of the Groups According to Exitus

	Group 1 (n = 33)	Group 2 (n = 117)	*P*
Age (years)	68.3 ± 11.8	59.7 ± 13.8	.002
Gender (male, n) (%)	19 (57.5)	68 (58.1)	.956
Length of stay in hospital (day)	15.8 ± 8.6	10.5 ± 7.2	<.001
Hypertension (n) (%)	20 (60.6)	52 (44.4)	.102
Diabetes mellitus (n) (%)	20 (60.6)	46 (38.5)	.026
Coronary artery disease (n) (%)	13 (39.4)	19 (15.4)	.003
Chronic pulmonary disease (n) (%)	3 (9.1)	13 (11.1)	.741
Chronic renal disease (n) (%)	11 (33.3)	13 (11.1)	.002
Systolic blood pressure (mm Hg)	125.2 ± 29.8	124.8 ± 19.7	.925
Diastolic blood presure (mm Hg)	64.0 ± 13.3	67.5 ± 11.9	.142
Heart rate (beat min^-1^)	93.5 ± 21.1	84.3 ± 14.3	.004
Temperature (°C)	37.9 ± 0.73	37.7 ± 0.90	.223
Respiratory rate (rate min^-1^)	22.3 ± 3.6	19.7 ± 3.2	<.001
Oxygen saturation (%)	82.7 ± 7.8	91.0 ± 7.0	<.001
Glucose (mg dL^-1^)	224.7 ± 129.2	153.7 ± 75.6	<.001
Urea (mg dL^-1^)*	108.7 (45.2-184.3)	31.0 (23.0-48.5)	<.001
Creatinine (mg dL^-1^)*	1.78 (0.91-3.62)	0.8 (0.63-1.04)	<.001
AST (U L^-1^)*	85 (41-144)	30 (24-47)	<.001
ALT (U L^-1^)	96.3 ± 165.6	31.8 ± 26.4	<.001
CRP (mg L^-1^)	188.6 ± 100.0	81.8 ± 83.1	<.001
D-dimer (mg L^-1^)*	4.4 (1.8-13.7)	0.79 (0.51-1.71)	.922
LDH (U L^-1^)	732.8 ± 1011.3	319.3 ± 170.5	<.001
Troponin I (pg mL^-1^)*	52.6 (16.1-215.7)	6.5 (2.8-13.5)	<.001
WBC (×10^9^ L^-1^)	16.8 ± 17.1	7.19 ± 4.01	.003
Platelet (×10^9^ L^-1^)	181.7 ± 98.6	240.8 ± 126.7	.015
Lymphocyte (×10^9^ L^-1^)	1.01 ± 0.72	1.36 ± 0.90	.044
Neutrophil (×10^9^ L^-1^)	15.0 ± 16.6	4.92 ± 2.79	<.001
NLR*	14.3 (4.6-25.9)	3.44 (2.16-6.41)	.011
Hydroxychloroquine (n) (%)	33 (100)	115 (98.2)	.595
Azithromycin (n) (%)	29 (87.9)	96 (82.1)	.498
Favipiravir (n) (%)	29 (87.9)	55 (47)	<.001
Lopinavir/ritonavir (n) (%)	12 (36.4)	24 (20.5)	.061

AST, aspartate transaminase; ALT, alanine transaminase; LDH, lactate dehydrogenase; CRP, C-reactive protein; WBC, white blood cells; NLR, neutrophil-to-lymphocyte ratio.

*Non-normally distributed variables were expressed with the median (25-75 percentile).

**Table 2.** Demographic Features, Vital Signs, Laboratory Findings, and the Medication of the Groups According to CCI Classification

	Mild CCI score (n = 28)	Moderate CCI score (n = 43)	High CCI score (n = 79)	*P*
Age (years)	46.3 ± 9.7	56.1 ± 10.3	70.1 ± 10.1	<.001
Gender (male, n) (%)	14 (50)	26 (60.4)	47 (59.5)	.635
Length of stay in hospital (day)	6.8 ± 4.1	11.2 ± 6.8	13.7 ± 8.5	<.001
Hypertension (n) (%)	4 (14.3)	17 (39.5)	51 (64.6)	<.001
Diabetes mellitus (n) (%)	2 (7.1)	15 (34.9)	49 (62)	<.001
Coronary artery disease (n) (%)	1 (3.6)	3 (7)	28 (35.4)	<.001
Chronic pulmonary disease (n) (%)	1 (3.6)	5 (11.6)	10 (12.7)	.399
Chronic renal disease (n) (%)	0 (0)	3 (7)	21 (26.6)	.001
Systolic blood pressure (mm Hg)	122.2 ± 11.6	122.8 ± 18.4	126.9 ± 26.6	.488
Diastolic blood presure (mm Hg)	67.0 ± 9.8	65.7 ± 12.1	67.2 ± 13.3	.797
Heart rate (beat min^-1^)	86.1 ± 17.3	85.3 ± 12.4	86.9 ± 18.1	.880
Temperature (°C)	37.8 ± 0.8	37.6 ± 0.9	37.9 ± 0.8	.107
Respiratory rate (rate min^-1^)	18.0 ± 2.4	20.6 ± 3.6	21.0 ± 3.3	<.001
Oxygen saturation (%)	93.5 ± 3.7	90.0 ± 8.5	87.2 ± 8.1	.001
Glucose (mg dL^-1^)	121.3 ± 67.0	152.5 ± 69.5	195.1 ± 105.4	.001
Urea (mg dL^-1^)*	23.6 (18.0-34.2)	29.0 (24.5-41.9)	56.5 (32.0-109.9)	<.001
Creatinine (mg dL^-1^)*	0.66 (0.57-0.81)	0.78 (0.61-0.89)	1.23 (0.82-2.2)	<.001
AST (U L^-1^)*	26.5 (23.2-34.4)	30.0 (25.0-58.0)	41.0 (27.0-85.0)	.156
ALT (U L^-1^)	29.6 ± 22.7	40.1 ± 31.9	55.0 ± 112.9	.342
CRP (mg L^-1^)	44.8 ± 52.6	79.8 ± 87.3	138.3 ± 101.3	<.001
D-dimer (mg L^-1^)*	0.87 (0.51-1.46)	0.64 (0.44-1.88)	1.86 (0.77-4.48)	.441
LDH (U L^-1^)	258.2 ± 87.2	329.7 ± 159.3	511.4 ± 701.7	.049
Troponin I (pg mL^-1^) *	2.7 (1.2-4.3)	4.6 (2.5-9.7)	19.7 (9.6-148.8)	.194
WBC (×10^9^ L^-1^)	5.58 ± 2.33	7.65 ± 4.76	11.53 ± 12.21	.007
Platelet (×10^9^ L^-1^)	222.0 ± 92.9	235.8 ± 124.1	225.5 ± 133.0	.874
Lymphocyte (×10^9^ L^-1^)	1.50 ± 0.81	1.25 ± 0.65	1.22 ± 0.99	.358
Neutrophil (×10^9^ L^-1^)	3.50 ± 1.38	5.14 ± 3.16	9.52 ± 11.80	.002
NLR*	2.40 (1.79-3.32)	3.72 (1.97-7.22)	5.35 (3.11-9.93)	.053
Hydroxychloroquine (n) (%)	27 (96.4)	43 (100)	79 (100)	.113
Azithromycin (n) (%)	23 (82.1)	38 (88.4)	64 (81)	.573
Favipiravir (n) (%)	3 (10.7)	25 (58.1)	56 (70.9)	<.001
Lopinavir/ritonavir (n) (%)	7 (25)	9 (20.9)	20 (25.3)	.856

AST, aspartate transaminase; ALT, alanine transaminase; LDH, lactate dehydrogenase; CRP, C-reactive protein; WBC, white blood cells; NLR, neutrophil-to-lymphocyte ratio; CCI, Charlson Comorbidity Index.

*Non-normally distributed variables were expressed with the median (25-75 percentile).

**Table 3.** Pearson’s Correlation Analysis Between CCI Score and Several Parameters

	*r*	*P*
Temperature	0.14	.092
Oxygen saturation	−0.37	<.001
Respiratory rate C-reactive protein D-dimer Lymphocyte WBC LDH Neutrophil-to-lymphocyte Troponin	0.34 0.42 0.14 −0.0650.32 0.23 0.091 0.15	<.001<.001.117 .420 <.001.005 .267 .090

WBC, white blood cells; LDH, lactate dehydrogenase; CCI, Charlson Comorbidity Index.

**Table 4.** Univariate and Multivariate Regression Analysis Showing the Parameters Related to Exitus

	Univariate			Multivariate		
OR	95% CI	*P*	OR	95% CI	*P*
Oxygen saturation (%)	0.882	0.836-0.932	<.001	0.888	0.816-0.965	.005
Heart rate (beat min^-1^)	1.031	1.008-1.054	.008	1.047	1.002-1.095	.040
Respiratory rate (rate min^-1^)	1.232	1.099-1.382	<.001			
Glucose (mg dL^-1^)	1.007	1.003-1.011	.001			
Urea (mg dL^-1^)	1.025	1.015-1.036	<.001	1.032	1.009-1.056	.006
Creatinine (mg dL^-1^)	2.307	1.576-3.377	<.001	0.324	0.137-0.767	.010
ALT (U L^-1^)	1.016	1.004-1.028	.009			
AST (U L^-1^)	1.035	1.020-1.050	<.001	1.034	1.012-1.056	.003
LDH (U L^-1^)	1.003	1.001-1.005	.005			
CRP (mg L^-1^)	1.011	1.007-1.016	<.001			
WBC (×10^9^ L^-1^)	1.168	1.086-1.255	<.001			
Platelet (×10^9^ L^-1^)	0.995	0.990-0.999	.017			
Lymphocyte (×10^9^ L^-1^)	0.462	0.277-0.938	.033			
Favipiravir (n)	8.173	2.702-24.716	<.001			
Charlson Comorbidity Score	1.831	1.466-2.288	<.001	1.990	1.314-3.015	.001

AST, aspartate transaminase; ALT, alanine transaminase; LDH, lactate dehydrogenase; CRP, C-reactive protein; WBC, white blood cells.
